# Inkjet-Printed Membrane for a Capacitive Acoustic Sensor: Development and Characterization Using Laser Vibrometer

**DOI:** 10.3390/s17051056

**Published:** 2017-05-06

**Authors:** Rubaiyet Iftekharul Haque, Erick Ogam, Patrick Benaben, Xavier Boddaert

**Affiliations:** 1 Centre Microélectronique de Provence (CMP), École Nationale Supérieure des Mines de Saint-Étienne, 13541 Gardanne, France; benaben@emse.fr; 2 Laboratoire de Mécanique et d’Acoustique UPR7051 CNRS, 4 impasse Nikola Tesla, CS 40006, 13453 Marseille Cedex 13, France; ogam@lma.cnrs-mrs.fr

**Keywords:** membrane, printed electronics, thin film, laser Doppler vibrometer

## Abstract

This paper describes the fabrication process and the method to determine the membrane tension and defects of an inkjet-printed circular diaphragm. The membrane tension is an important parameter to design and fabricate an acoustic sensor and resonator with the highest sensitivity and selectivity over a determined range of frequency. During this work, the diaphragms are fabricated by inkjet printing of conductive silver ink on pre-strained Mylar thin films, and the membrane tension is determined using the resonant frequency obtained from its measured surface velocity response to an acoustic excitation. The membrane is excited by an acoustic pressure generated by a loudspeaker, and its displacement (response) is acquired using a laser Doppler vibrometer (LDV). The response of the fabricated membrane demonstrates good correlation with the numerical result. However, the inkjet-printed membrane exhibits undesired peaks, which appeared to be due to defects at their boundaries as observed from the scanning mode of LDV.

## 1. Introduction

The membrane is an important mechanical basic element in microelectronics, especially in the field of acoustic microelectromechanical systems (MEMS) devices. Among the different types of membranes, circular membranes are the most commonly used due to their large number of applications, namely musical instruments, condenser microphones, hearing aids, etc. [1,2].

Resonant systems are highly preferable for several interesting applications, such as navigation of drones and autonomous system or bio-mimetic development based on animal instincts [3]. Study shows that insects, like crickets, can navigate using sound sources in complex environments. The same principle can be explored to fabricate autonomous micro-flying objects [3]. Since the acoustic resonant sensor can provide good sensitivity and selectivity without the use of any passive and electronic filters [4], it can be an ideal candidate for such bio-mimetic applications. The frequency of such a transducer can be tuned by controlling the membrane tension or by changing the dimension of the device.

Generally, photolithography, a subtractive method, and vacuum deposition techniques, such as, physical vapor depositions, chemical vapor deposition, are used for MEMS fabrications. Although well established, photolithographic patterning involves various steps, exhibits higher production cost, processing time and generates large volumes of hazardous waste. In addition, the vacuum deposition techniques that are used to deposit the conductive layer are energy intensive and require additional masking step to create the pattern [5]. These problems can be overcome by employing printing techniques to develop electronic components and devices without the etching step. Printing techniques offer simple fabrication steps along with reduced logistic costs, large area production and less energy consumption. In addition, the printed electronics procedures generally require relatively lower processing temperature in atmospheric conditions. Thus, printed electronics enable the production of electronic components on inexpensive, lightweight, thin and flexible substrates [6,7]. Consequently, compared to the conventional electronics, printed electronics exhibit increased robustness, and generally withstand heat, rough handling and harsh environmental conditions more effectively [8,9,10].

Inkjet printing, amongst the well-developed printing methods, is a contact-free additive technique that deposits droplets of liquid material with high precision onto a substrate at room temperature in ambient conditions. Furthermore, inkjet printing involves the use of fewer hazardous chemical. As there is no requirement of masks or screens, this technique is more flexible, versatile and can be setup with relatively low effort [5,11,12]. The inkjet printing technique is limited to the pattern direct-printing of the base layer and the subsequent curing of the deposited ink to remove solvents, to initiate the film cohesion and to improve the functionality of the printed layer. However, stable and optimally-designed ink is required for inkjet printing. Improperly-designed ink either prevents drop ejection due to dissipating pressure pulse in the ink or forms satellite drops rather than a single droplet. Moreover, often a non-homogeneous layer thickness formed because of the coffee-ring effect during the drying process [13,14]. Generally, the conventional oven sintering method or other selective techniques, such as laser, microwave, joule heating [15,16,17,18] and the photonic sintering technique [5,19,20], can be used for the curing step. Inkjet printing is generally employed to manufacture electrical interconnects and different embedded electrical passive structures, namely resistors, capacitors, radio frequency identification (RFID) antennas and even to develop under-bump metallization [21] and active devices, like, organic field effect transistors and light-emitting films [14]. In addition, 3D MEMS structures [22,23] can also be manufactured using the inkjet printing technique [22,23]. However, this method still requires the removal of the sacrificial layer, and the structures are mechanically weak. These issues can be addressed by developing a diaphragm by inkjet printing of a conductive layer on pre-strained thin polymer film [4].

Recently, a new concept of fabricating a capacitive acoustic resonator has been presented using the inkjet printing technique [4]. To achieve good sensitivity and selectivity of the capacitive resonator, combining numerical simulation and design of experiment [24], optimization of six device parameters, namely membrane radius, back plate radius, cavity height, air gap, membrane tension and membrane thickness, is required. Amongst them, membrane tension is one of the most important parameters to tune the resonator.

Different materials are used to fabricate membranes [25], and their thickness is typically in the range of 0.5 μm to 500 μm [26]. Although metals are the most suitable for diaphragm fabrication due to their electrical properties, they are less resistive to corrosion during their lifetime. In addition, as metals are vulnerable to the aggressive thermal and chemical environment, it creates problems to work with metals during the device manufacturing process. Thus, the membranes for most of the MEMS are often manufactured using silicon substrates using conventional photolithographic patterning and surface micromachining techniques. Occasionally, diaphragms for the transducers have also been fabricated using support membrane made of chemically more robust organic thin films having the desired mechanical properties [27].

Due to complex boundary conditions, shapes and applications, the vibration of membranes remains an active subject of research [1]. The accuracy of membrane strain and the behavior of the membrane under the sound pressure drive the response and sensitivity of the acoustic sensor. Study shows that the laser vibrometer technique allows determining the membrane tension based on the measurement of its resonant frequency [28].

At resonant frequency, which is known as the normal mode of vibration, maximum membrane displacement is observed because of the sinusoidal vibration of all parts of the membrane with the same frequency and with a fixed phase relation. In a vacuum, resonant frequencies of the membrane are influenced by its physical dimensions and mechanical constants, such as Young's modulus, density of the membrane material ($\rho_{m}$), radius of the membrane ($R_{m}$), membrane tension ($T_{m}$) and boundary conditions. The natural frequencies (in a vacuum) of the pre-tensed circular vibrating membrane can be expressed as follows [29,30],

(1) $$f_{ij}=\frac{k_{ij}}{2\pi R_{m}}\sqrt{\frac{T_{m}}{t_{m}\rho_{m}}}=\frac{k_{ij}}{2\pi R_{m}}\sqrt{\frac{T_{m}}{\rho_{ms}}}$$

The values $k_{ij}$ are deducted from the roots of the Bessel functions of the first kind, and the values of $k_{ij}$ for the first six modes are listed in Table 1. The natural frequencies of vibration and mode shapes are defined by two integers (i,j), where the index i=1, 2, 3, … corresponds to the number of circumferential lines (r=constant) on the membrane that have zero displacement, and the index j=0, 1, 2, … corresponds to the number of diametral lines (θ=const.) that have zero displacement. $\rho_{ms}=\rho_{m}t_{m}$ represents the surface density of the membrane material.

Therefore, the tension ($T_{m}$) of the membrane can thus be calculated from Equation (1), as follows:

(2) $$T_{m}=t_{m}\rho_{m}{\left(\frac{2\pi f_{ij}R_{m}}{k_{ij}}\right)}^{2}$$

In this work, the fabrication process of the membrane using the inkjet printing technique is presented, and a simple and easy method to determine the membrane tension and to identify the defects using laser Doppler vibrometer is discussed.

## 2. Experimental Details

### 2.1. Materials and Printing System

Thin polyethylene terephthalate (PET) film, also known as Mylar film, having thicknesses of 8 μm and 23 μm that are used as the support membrane, were purchased from Technifilm Company (Valence, France). The electrode on thin polymer film has been printed using Suntronics silver ink U5714 having 40 wt % of metal content from SunChemical (Parsippany, NJ, USA). Prior to the printing, the surface of the substrate was rinsed with isopropanol and dried using blown nitrogen. During this work, a Dimatix DMP-2800 material inkjet printing system from Fujifilm (Santa Clara, CA, USA) with 10 pL cartridges consisting of 16 nozzles was used to perform printing. Table 2 summarizes the printing parameters used during this work.

### 2.2. Fabrication

**Figure 1:**
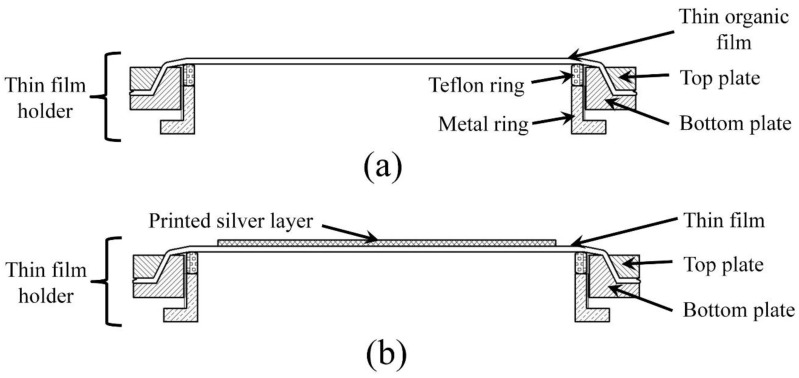
Schematic diagram of the cross-sectional view of the membrane fixed on the thin film holder (**a**) before and (**b**) after printing and sintering of the conductive layer.

The diaphragm fabrication was performed by depositing a conductive layer on pre-strained thin polymer film using the inkjet printing technique. During this process, tension was applied at the periphery of the thin organic film, mounted on a specific designed holder. Afterward, the conductive layer was printed on the pre-strained film and sintered at 140 °C for 30 min using a conventional thermal heat oven. Figure 1a,b illustrates the printing process of the conductive layer on pre-strained thin polymer film.

**Figure 2:**
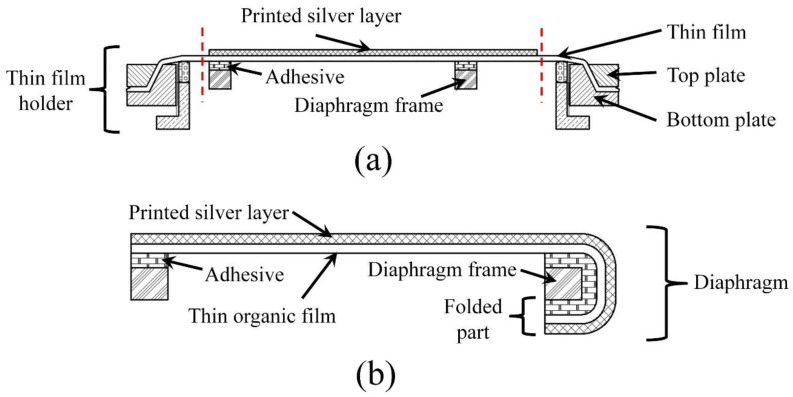
Schematic diagram of the cross-sectional view: (**a**) after gluing the diaphragm frame to the thin film with the printed electrode fixed on the thin film holder; (**b**) final diaphragm after separating from the large film holder.

**Figure 3:**
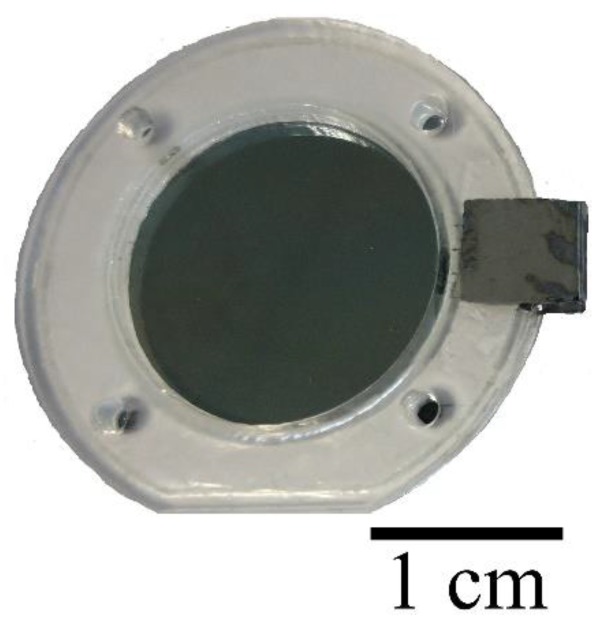
Photograph of the inkjet-printed diaphragm fixed on the diaphragm frame.

Adhesive was then used to attach a rigid diaphragm frame on the thin organic film, opposite to that of the printed conductive layer. Thereafter, the diaphragm was cut off from the film holder (Figure 2a), and as illustrated in Figure 2b, the extended part of thin film with the conductive layer was folded in one portion and glued to the opposite surface of the frame to obtain electrical connection from both directions. Figure 3 presents the printed circular diaphragm. More details about printing of the conductive layer on thin Mylar film can be found in [4].

### 2.3. Experiments

**Figure 4:**
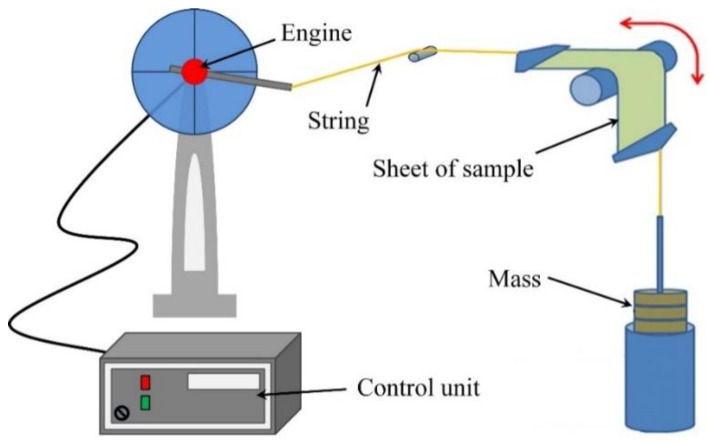
Schematic diagram of the experimental setup of the bending fatigue machine.

Initially, the mechanical performances, such as, the adhesion, the flexibility and the lifecycle, of the printed conductive silver layers on thin Mylar film, have been evaluated. In this regard, the bending test has been performed on the square-shaped inkjet-printed conductive silver layers on the thin film, sintered at 140 °C for 30 min. Prior to the annealing process, printer layers were kept at rest at room temperature for 15 min, which stabilized the printed layer. During the bending test, curvature radii of 5 mm and 25 mm were used under constant tension. Thereafter, their electrical properties were measured using a JANDEL (model: RM3-AR, Linslade, UK) four-point probing resistance measurement system, and microstructures were investigated by optical microscope (model: Eclipse L200 from Nikon, Tokyo, Japan). The experimental setup of the bending test is illustrated in Figure 4. The thicknesses of the printed conductive layers were measured using a mechanical profilometer (model: XP-2, AMBiOS Technology, Santa Cruz, CA, USA).

**Figure 5:**
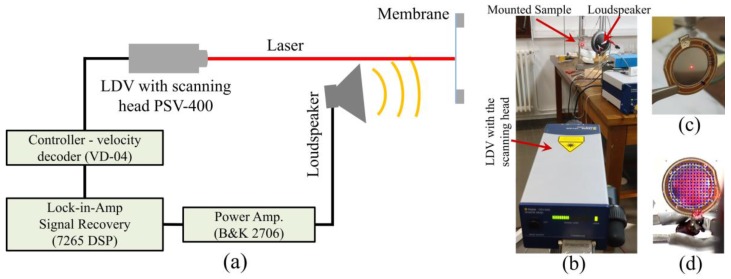
The experimental setup for the laser Doppler vibrometer (LDV) measurement: (**a**) schematic diagram; (**b**) photograph of the setup; (**c**) position of the laser spot at the center of the membrane for single-point mode measurement; and (**d**) the grid used for the laser during the scanning mode measurement.

To understand the dynamic characteristics of the diaphragm, non-contact laser Doppler vibrometer (LDV, model: Polytec PSV-400 Scanning laser Vibrometer (Waldbronne, Germany)) under acoustic pressure harmonic excitation between 20 Hz and 20 kHz has been employed. A loudspeaker (B&C-DE-700-8, Florence, Italy) was used to generate acoustic pressure excitation, and LDV was used to record the corresponding responses in single-point mode and scanning mode. The experimental setup of the LDV measurement is illustrated in Figure 5. In single-point mode, the laser spot remains fixed at the center position of the membrane to acquire the response spectrum. On the other hand, during scanning measurement mode, the laser spot scans through the defined points on the surface of the membrane to acquire the surface deflection modes. At the end, the experimental results were compared with the numerical results obtained using COMSOL Multiphysics software (Version 5.1).

## 3. Results and Discussions

### 3.1. Conductive Layer Printing on Thin Film

The stable ink and the stable droplet formation during printing along with the post-printing process led to the reduction of the coffee-ring effect and provided uniform layers. Furthermore, electrodes were patterned with marginally larger area than the boundary of the frame to keep the non-homogeneous coffee-ring section, if there was any, outside of the active membrane zone.

As the conductivity of the membrane is one of the important factor for practical applications. Initially, the four-point probing resistance measurement system has been used to measure the sheet resistance (*R_sq_*) of the printed silver layer on thin film, and then, the resistivity (*ρ*) is calculated from Equation (3).

(3) $$\rho =R_{sq}\cdot t$$

where *t* refers to the thickness of the conductive layer. In this case, the measured thickness of the printed silver layer on Mylar film is 550 ± 50 nm. Finally, the electrical conductivity (*σ*) in S/m, which is the reciprocal of the resistivity, is calculated using the following expression,

(4) $$\sigma =\frac{1}{\rho }=\frac{1}{R_{sq}\cdot t}$$

**Figure 6:**
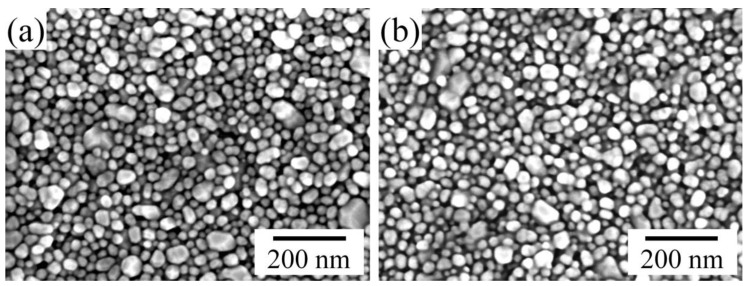
Scanning electron microscope images of silver nanoparticles after (**a**) drying at 90 °C for 30 min; and (**b**) sintering at 140 °C for 30 min.

Electrical measurements have shown that the conductivity of the printed silver layer sintered at 140 °C for 30 min is 1.4 × 10^7^ S/m ± 0.1 × 10^7^ S/m compared to the conductivity of bulk silver of 6.3 × 10^7^ S/m [31,32,33]. The achieved conductivity of the printed silver layer is satisfactory, and can be explained by the neck formation between the nanoparticles due to material transfer between them during sintering. The microstructures of the sintered printed silver layers after drying at 90 °C and sintering at 140 °C for 30 min, respectively, are presented in Figure 6.

### 3.2. Bending Test

**Figure 7:**
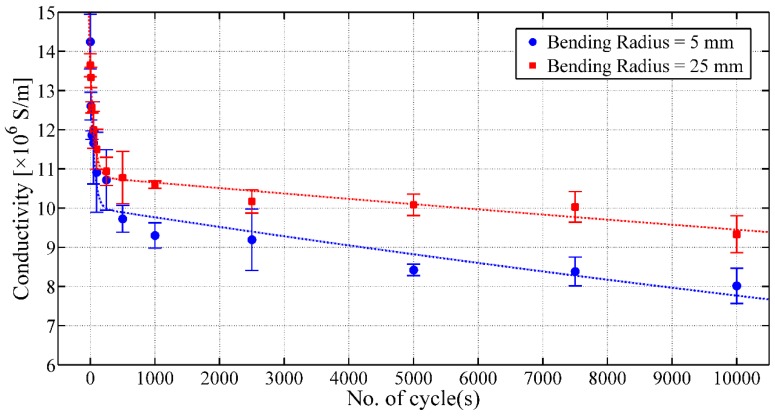
Conductivity of the inkjet-printed conductive silver layer on thin Mylar film versus the number of bending cycles.

**Figure 8:**
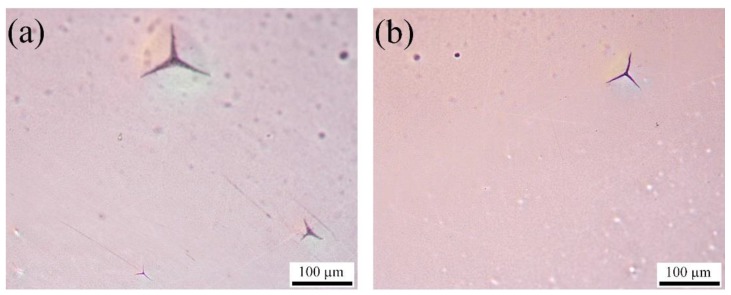
Optical viewgraph of the inkjet-printed conductive silver layers on thin Mylar film after the bending test for 10,000 cycles: (**a**) bending radius of 5 mm; and (**b**) bending radius of 25 mm.

It has been observed that the printed layers were mechanically stable and attached to the thin polymer film. However, a decrease in conductivity of the printed silver layers is noticed with the increasing number of bending cycles (Figure 7). After 10,000 cycles, the conductivity is 8.0 × 10^6^ S/m ± 0.5 × 10^6^ S/m and 9.3 × 10^6^ S/m ± 0.5 × 10^6^ S/m for bending radii of 5 mm and 25 mm, respectively. The initial sharp decrease of the conductivity of printed silver layers might be due to the initial stretching of the thin polymer film due to the applied load, which is stabilized after 250 cycles. This increase in resistance is also caused by the formation of local small micro-cracks on the printed layers due to stretching and bending, as illustrated in Figure 8.

Experiment has revealed that the larger bending radius, compared to the smaller bending radius, produces less localized cracks and, thus, causes less degradation of the electrical properties for a higher number of bending cycles. However, the overall deterioration of the electrical properties due to bending remains within a good applicable conductivity range. Thus, from the bending test, it can be concluded that the adhesion of the printed silver layer on thin Mylar film is very stable and strong, and the delamination of the printed silver layers does not occur even after large bending or deformation. Therefore, these printed membranes exhibit robustness and will provide longevity of the MEMS devices.

### 3.3. Acoustic Characterization of the Membrane Using Laser Doppler Vibrometer

**Figure 9:**
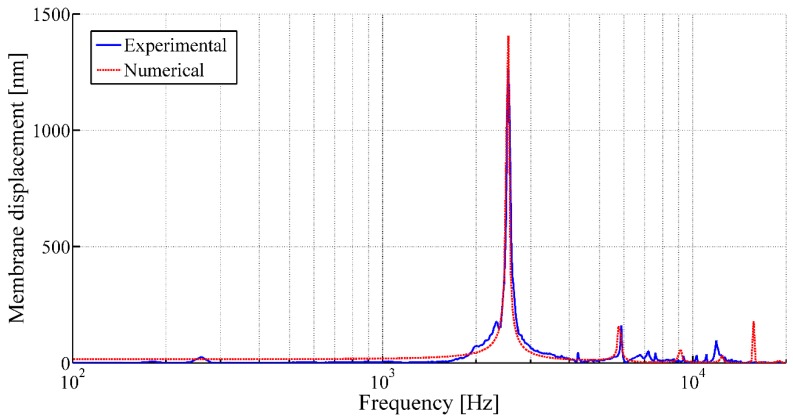
Frequency response of the circular membrane with a membrane radius of 10 mm and a membrane thickness of 8 µm under acoustic pressure (using LDV in single-point mode).

**Figure 10:**
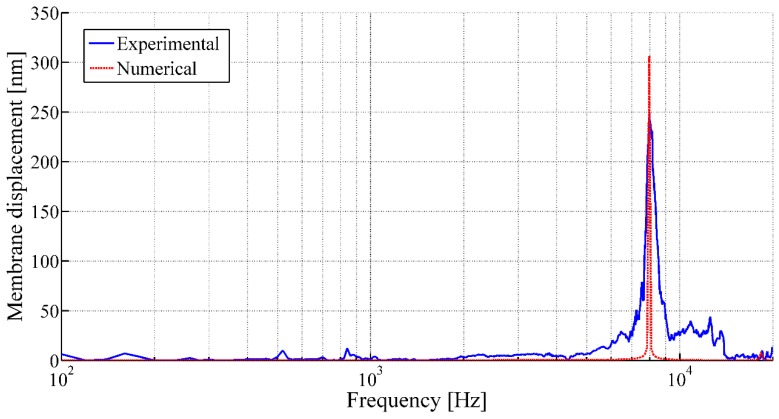
Frequency response of the circular membrane with a membrane radius of 4 mm and a membrane thickness of 8 µm under acoustic pressure (using LDV in single-point mode).

The vibrational behaviors of the membranes are studied using LDV in single-point mode. Figure 9 and Figure 10 illustrate the frequency responses of the membranes having a membrane radius of 10 mm and 4 mm, respectively, for a membrane thickness of 8 µm along with the numerical simulation results.

The acoustic characterizations of the membranes reveal sharp rise in membrane displacements at certain frequencies under acoustic excitation, which refers the membrane displacements at first resonance frequencies of the corresponding membranes (Figure 9 and Figure 10). Thereafter, the tension of the membranes ($T_{m}$) are calculated from the measured resonant frequencies ($f_{ij}$) using Equation (2). The experimental membrane tensions based on the first resonant frequencies from LDV measurement along with the numerical analysis results are tabulated in Table 3.

**Figure 11:**
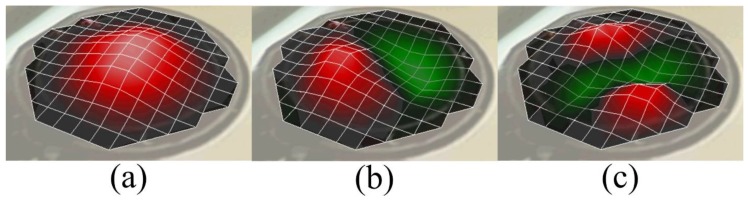
Different modes of the vibrations of the membrane with a membrane radius of 10 mm and a membrane thickness of 8 µm using pre-tension Mylar thin film observed using LDV in scanning mode: (**a**) Mode (1,0) at 2540 Hz; (**b**) Mode (1,1) at 4030 Hz; and (**c**) Mode (1,2) at 5505 Hz.

It has been observed that, for a constant membrane thickness, with the increasing membrane radius, the resonance moves towards the lower frequencies for the same initial tension at their periphery. On the other hand, for a constant membrane radius with the increasing membrane thickness, higher membrane tension at its periphery has to be applied to achieve similar first resonant frequency. In addition, different vibration mode shapes of the membrane in air can be observed using LDV in scanning mode. Figure 11 presents different mode shapes as observed for the circular membrane having a radius and thickness of 10 mm and 8 μm, respectively.

**Figure 12:**
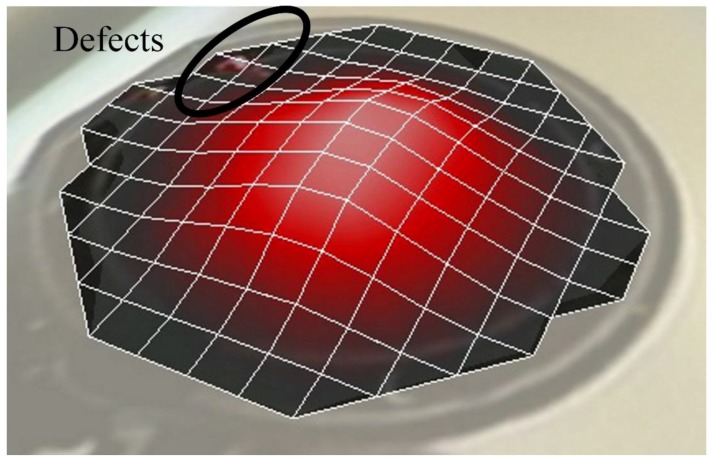
Observation of the defects on the fabricated printed membrane with a membrane radius of 10 mm and a membrane thickness of 8 µm using pre-tension Mylar thin film using LDV in scanning mode.

**Figure 13:**
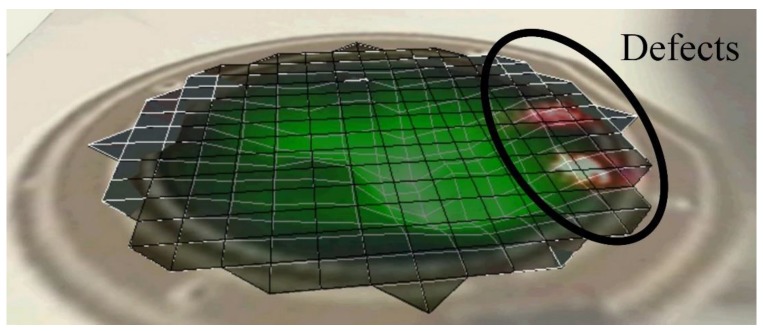
Observation of the defects on the fabricated printed membrane with a membrane radius of 4 mm and a membrane thickness of 8 µm using pre-tension Mylar thin film using LDV in scanning mode.

Compared to the numerical analysis, the experimental response of these membranes often shows undesired peaks and inconsistency, as observed from Figure 10, which could be related to the appearance of defects during fabrication. It has been demonstrated that the LDV study in scanning mode allows highlighting the defects of the membranes, as well. These defects generally arise at the boundary of the membrane and could be related to a gluing problem of the pre-tensed film with the frame. The defects may have caused non-linearity in the membrane tension and thus disturb the membrane behavior. The scanning mode observation of membranes having a membrane radius of 10 mm and 4 mm, respectively, for a membrane thickness of 8 µm fixed at its periphery, as illustrated in Figure 12 and Figure 13, have confirmed the hypothesis. The observations from the scanning mode of LDV, are in good agreement with the membranes’ behaviors. The membrane with smaller defects (Figure 12) exhibits similarities with the numerical results (Figure 9), whereas the membrane with higher amount boundary defects (Figure 13) showed more divergence compared to the numerical result (Figure 10).

## 4. Conclusions

The fabrication of the inkjet-printed membrane and the technique used to determine its tension and defects are discussed. As presented, inkjet printing could be an alternative new way to fabricate cost-efficient, flexible and robust membranes for MEMS devices. The printed conductive layer exhibits good bonding with the substrate and stable electrical properties with minor deterioration over long bending cycles. The laser vibrometer technique, a non-contact approach, allows the determination of the membrane tension precisely. As expected, the peak of the first resonant frequency shifts to the higher frequencies for the smaller membrane size for fixed membrane thickness. However, the fabricated membrane occasionally exhibits defects due to a gluing problem at its periphery that may induce non-linearity in membrane tension and thus affect its response. The present study also confirms that defects at the boundaries of the membrane can be observed using LDV in scanning mode. Unfortunately, there was a lack of appropriate adhesive to hold Mylar film under high strain with the frame. The gluing problem might be solved by using the plastic welding with laser technique [34]; however, this required more in depth study of plastic welding and joining.

Therefore, the non-contact measurement and characterization method using a laser-vibrometer is an appropriate technique to study the membrane behavior and to determine the membrane tension. This method could also be used to detect physical defects in scanning mode. Such a kind of printed membrane can be used to fabricate capacitive acoustic sensors and resonators [4].

## Figures and Tables

**Table 1:** Values of $k_{ij}$ deducted from the roots of the Bessel functions of the first kind for the first six modes [24].

Mode Number	Factor
1	$k_{10}=2.4048$
2	$k_{11}=3.8317$
3	$k_{11}=3.8317$
4	$k_{12}=5.1356$
5	$k_{12}=5.1356$
6	$k_{20}=5.5201$

**Table 2:** Printing parameters.

Parameter	Value
Drop spacing	25 µm
Firing voltage	28 V
Jetting frequency	13.3 kHz
Nozzle temperature	38 °C
Substrate temperature	Room temperature
Meniscus vacuum	4.5 inches H_2_O or 1120.9 Pa
Cartridge height	0.8 mm
No. of nozzles used	9

**Table 3:** Calculated membrane tension of different membrane sizes from the first resonant peak.

Membrane		Experimental Result		Numerical Result	
Radius ($R_{m}$)	Thickness ($t_{m}$)	First resonant frequency ($f_{10}$)	Calculated membrane tension ($T_{m}$)	First resonant frequency ($f_{10}$)	Calculated membrane tension ($T_{m}$)
mm	μm	Hz	N/m	Hz	N/m
4	8	7980	77.34	7963.4	77.0
4	23	7310	186.3	7259.4	184.0
6	8	4780	62.4	4786.3	62.6
6	23	3880	118.3	3856.6	116.9
8	8	3840	71.6	3856.6	72.3
8	23	3330	154.9	3305.2	152.6
10	8	2540	49	2542.7	49.1
10	23	2730	162.6	2704.6	159.6
